# Swedish Web Version of the Quality of Recovery Scale Adapted for Patients Undergoing Local Anesthesia and Peripheral Nerve Blockade (SwQoR-LA): Prospective Psychometric Evaluation Study

**DOI:** 10.2196/23090

**Published:** 2021-01-15

**Authors:** Ulrica Nilsson, Karuna Dahlberg, Maria Jaensson

**Affiliations:** 1 Department of Neurobiology, Care Sciences and Society Karolinska Institutet Huddinge Sweden; 2 Perioperative Medicine and Intensive Care Karolinska University Hospital Stockholm Sweden; 3 School of Health Sciences Faculty of Medicine and Health Örebro University Örebro Sweden

**Keywords:** day surgery, local anesthesia, peripheral nerve blockade, postoperative recovery, psychometric evaluation

## Abstract

**Background:**

The frequency and timing of assessing patient symptoms and discomfort during postoperative recovery are goals. Therefore, real-time recovery evaluation has been suggested to identify specific deficits in patient recovery.

**Objective:**

This study aimed to psychometrically evaluate the Swedish Web Version of the Quality of Recovery (SwQoR) Scale adapted for patients undergoing local and peripheral nerve block (SwQoR-LA).

**Methods:**

This was a secondary analysis of a psychometric evaluation of 107 patients aged ≥18 years undergoing day surgery under local or peripheral nerve block anesthesia at 4 different day surgery departments in Sweden. The SwQoR-LA, available through a mobile app called Recovery Assessment by Phone Points (RAPP), was completed daily on postoperative days 1-7.

**Results:**

Some evidence of construct validity was supported, and discriminant validity was found in 7 of 8 items related to general anesthesia. The internal consistency was acceptable (.87-.89), and the split-half reliability was 0.80-0.86. Cohen d effect size was 0.98, and the percentage of change from baseline was 43.4%. No floor nor ceiling effects were found.

**Conclusions:**

The SwQoR-LA is valid, reliable, responsive, and clinically feasible for digital real-time recovery assessment of patient recovery to identify specific deficits in patient recovery and detect those patients who might beneﬁt from a timely intervention.

**Trial Registration:**

ClinicalTrials.gov NCT02492191; https://clinicaltrials.gov/ct2/show/NCT02492191

**International Registered Report Identifier (IRRID):**

RR2-10.1136/bmjopen-2015-009901

## Introduction

Postoperative recovery is an individual process and a transformative journey to a new stable state [1]. It has a clear starting point [1], followed by a dynamic and individual process including physical, psychological, social, and habitual dimensions [1-3] that affect each other [1]. Due to advances in surgery, anesthesia, nursing care, and early mobilization, postoperative outcome and recovery have improved [4,5]. Inpatient surgery has decreased in favor of day surgery. Postoperative care at the hospital for day surgery patients is short, as the patients are discharged on the same day or within 24 hours [6]. This quick discharge implies that patients must take on great responsibility for their recovery process [7,8]. It is extremely important to follow up with and support patients in their recovery, both at the hospital and after discharge. Hence, several different patient-reported outcome questionnaires have been developed and tested and are recommended for use in clinical practice and clinical trials in surgery and anesthesia [9-11]. However, the frequency and timing of such assessment must be considered, and measurement at a single time point can be highly problematic [12]. Therefore, real-time recovery evaluation—that is, the simultaneous collection, analysis, and reporting of data occurring at different clinically relevant postoperative intervals—has been suggested to identify specific deficits in patient recovery [12-14].

To our knowledge, only one evidence-based questionnaire has been adapted for daily assessment for measuring patient-reported postoperative symptoms through an electronically assessed follow-up questionnaire: the Swedish web version of Quality of Recovery (SwQoR). The SwQoR questionnaire has been made available through an app called Recovery Assessment by Phone Points (RAPP) and includes 24 postoperative symptoms related to surgery and anesthesia [15-17]. Psychometric evaluation of the SwQoR has been performed and revealed high validity and reliability and a high degree of responsiveness; thus, the SwQoR was found to be clinically feasible for use in the systematic follow-up of patient postoperative recovery [18].

Based on experience from day surgery departments using RAPP in clinical practice, a short form for patients who have undergone day surgery under local anesthesia or peripheral nerve block has been requested. Some of the symptoms included in the SwQoR are related to general anesthesia and could therefore be excluded for the questionnaire to be more user-friendly for this group of patients. After discussion with the staff at the day surgery departments and based on our own experience, 8 symptoms related to general anesthesia were deleted from the SwQoR: sore throat, sore mouth, voice not sounding the same as usual, having trouble breathing, muscle pain, trouble urinating, diarrhea, and feeling constipated. The aim of this study was to undertake a psychometric evaluation of the real-time recovery questionnaire SwQoR-LA after adapting it for patients undergoing local anesthesia and peripheral nerve block.

## Methods

### Study Design

This study involved a psychometric evaluation of data originating from a multicenter, 2-group, parallel, single-blind, randomized controlled trial conducted from October 2015 to July 2016 at 4 day surgery departments in Sweden. The primary aim was to estimate the cost-effectiveness of using, vs not using, RAPP for follow-up on recovery after day surgery [19]. This study involves only those participants who were randomized into the intervention group and who underwent local or peripheral block anesthesia. This study followed the ethical standards of the Helsinki Declaration (6th revision) and was approved by the Uppsala Regional Ethics Committee (2015/262).

### Sample

The data collection procedure was as follows. Information on the planned surgery was provided to the patients together with written information on the study. Upon their arrival at the day surgery department, a research nurse provided patients with oral information about the study and invited them to enroll. The inclusion criteria were age ≥18 years, undergoing day surgery, able to understand written and spoken Swedish, and access to a smartphone. Exclusion criteria were memory and visual impairment, undergoing surgical abortion, and ongoing substance abuse.

### SwQoR-LA

The SwQoR-LA includes 16 of the 24 postoperative symptoms included in the SwQoR. The symptoms are scored on an 11-point numeric visual analogue scale from 0 (“none of the time”) to 10 (“all of the time”). Each question appears separately on the screen, and a dot on the visual analogue scale has to be moved to indicate an answer. The symptoms disappear from the screen immediately after a response is given, and each question on a symptom must be answered to submit the daily assessment [20].

### Procedures

Preoperatively, the research nurse assisted with the installation of RAPP, including SwQoR, onto each participant’s smartphone for both participants who underwent general and local or peripheral block anesthesia. The participants were encouraged to do a test run of the app by putting in fake responses. The research nurse also explained other functionalities of the RAPP, such as how to move between the items and how to use the navigation keys.

The participants were instructed to complete the SwQoR in the RAPP every day until postoperative day 14. A daily reminder helped the participants to remember to send in their daily report on their recovery. The health care professionals at the day surgery department had access to all patient data via a web administrator interface.

This study includes data for the 16 symptoms (ie, SwQoR-LA) on postoperative days 1-7 from the participants that underwent local or peripheral block anesthesia. Based on the opinion of both patients and clinicians using RAPP in clinical practice, 7 days of assessment was considered appropriate, as a short recovery period after minor surgery with local or peripheral block anesthesia is expected. In addition to the SwQoR, other collected variables were age, gender, American Society of Anesthesiologists physical status, and type of anesthesia.

### Psychometric Evaluation

The psychometric evaluation was guided by the COnsensus-based Standards for the selection of health Measurement INstruments (COSMIN) [21] and a previous psychometric evaluation of the SwQoR [18]. Acceptability, which measures the clinical user friendliness, was assessed in terms of the successful response rate on postoperative days 1-7. Floor and ceiling effects (ie, the number of respondents who achieved the lowest or highest possible scores) were measured on days 1-7; it was considered a problem if more than 15% of the study population achieved the lowest or highest possible score [22]. Construct validity is the extent to which questionnaire scores are consistent with hypotheses, assuming that the questionnaire validly measures the construct being addressed. A correlation coefficient >0.4 was considered to be evidence of construct validity (ie, moderate to strong correlation). To analyze construct validity, a priori hypothesis testing was conducted, under the hypothesis that the SwQoR-LA, just as with the SwQoR [18], on day 1 would correlate positively with the duration of surgery, duration of stay at the postanesthesia care unit (PACU), and patient age. In addition, lower quality of recovery (ie, higher degree of postoperative symptoms) was not expected in women versus men, just as with the SwQoR [18]. Discriminant validity was tested on day 7, and it was expected that patients who underwent local anesthesia would have significantly lower scores on the symptoms related to general anesthesia that are not included in the SwQoR-LA: voice not sounding the same as usual, sore throat, sore mouth, having trouble breathing, muscle pain, trouble urinating, diarrhea, and feeling constipated. For example, sore throat and sore mouth are symptoms related to the endotracheal tub or laryngeal mask used under general anesthesia.

Reliability was assessed with (1) internal consistency, by measuring the average correlation between the SwQoR items on days 1-7, indicated by Cronbach α, and (2) split-half reliability, by measuring the correlation between randomly split segments of the SwQoR on days 1-7. Responsiveness, which was used to evaluate the SwQoR-LA’s sensitivity and ability to detect clinically important changes, was measured with (1) Cohen *d* effect size, calculated as average changes in scores from days 1 to 7, divided by the pooled SD of all measurements (where 0.2-0.5 indicates a small effect, 0.5-0.8 a moderate effect, and 0.8-1.2 a large effect) [23], and (2) mean changes over time and percent changes from baseline on days 1-7.

### Statistical Analysis

The sample size was calculated for the original randomized controlled trial [19]; therefore, no sample size was calculated for the SwQoR-LA. Descriptive statistics are presented as means, SDs, numbers and percentages, ranges or minimum-maximum, or 95% CI for the sake of clarity. In this study, when analyzing the overall level of recovery after local anesthesia, we used the global score of the SwQoR-LA, with a minimum value of 0 and a maximum value of 160.

To investigate differences between symptoms and gender, the Mann-Whitney U-test was performed. Associations were measured with Spearman rank coefficients (rho). Cronbach α and split-half reliability with the Spearman-Brown coefficient were used to assess the internal consistency. SPSS version 24 (IBM Corp, Armonk, NY) for Windows was used for the statistical analyses. The null hypothesis was rejected at a two-tailed *P*<.05.

## Results

### Acceptability

Of the 513 patients, 19 were excluded due to cancelled operations (n=15), refusal to participate (n=3), or technical issues (n=1), leaving 494 patients. Of the remaining patients, 107 underwent local or peripheral nerve block anesthesia, 362 underwent general anesthesia, and 25 had missing information about the type of anesthesia and were thereby excluded from the analysis. The results of this study only include the patients who underwent local anesthesia (n=107), except for the discriminant validity analysis. Patients’ demographic variables and perioperative factors are presented in Table 1.

The response rate was 88.8% (95/107) on postoperative day 1 and 72.9% (78/107) on day 7. The global SwQoR-LA score decreased from 35.7 (SD 24.4) on day 1 to 15.5 (SD 15.5) on day 7 (Table 2).

**Figure 1:**
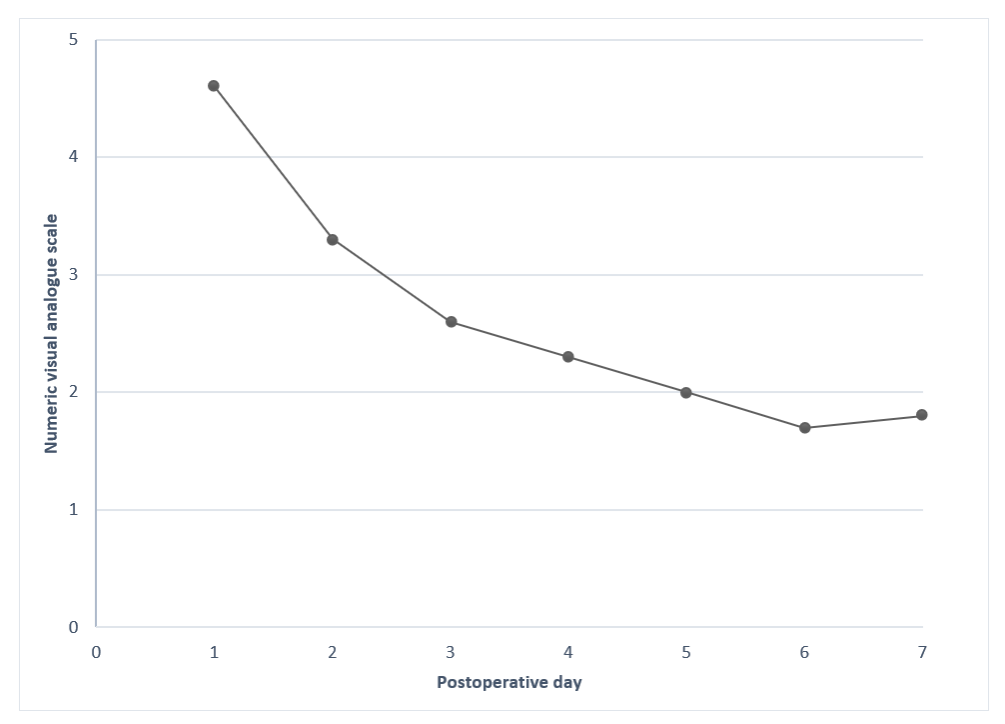
Pain in the surgical wound on postoperative days 1-7.

Because the patients had to respond to each item in order to move on to the next item, there were no missing answers. Pain in the surgical wound was the symptom that occurred most frequently, starting with a value of 4.6 on day 1 and ending at 1.8 on day 7 (Figure 1).

**Table 1:** Demographic variables and surgical and anesthetic factors (n=107).

Variables		Values
**Gender n (%)**		
	Male	35 (33)
	Female	72 (67)
Age (years), mean (SD)		49 (14)
Age (years), median (minimum-maximum)		55 (18-73)
**ASA^a^, n (%)**		
	I	31 (29)
	II	19 (18)
	Missing information	57 (53)
**Type of anesthesia, n (%)**		
	Local infiltration	66 (62)
	Intravenous regional anesthesia (IVRA)	24 (22)
	Sciatic nerve block	17 (16)
**Type of surgery, n**		
	Orthopedics	46
	Hand	39
	General	8
	Ear, Nose, and Throat (ENT)	8
	Gynecology	3
	Urology	2
	Dental	1
Duration of surgery (minutes), mean (SD)		34 (25)
PACU^b^ stay (minutes), mean (SD)		82 (53)

^a^ASA: American Society of Anesthesiologists.

^b^PACU: postanesthesia care unit.

**Table 2:** Mean and range of the symptom scores on postoperative day 1 (n=95).

Item	Symptom score	
	Mean (SD)	Minimum-maximum
Sleeping difficulties	2.1 (2.8)	0-10
Not having a general feeling of well-being	2.9 (2.8)	0-9
Not feeling in control of my situation	2.4 (2.9)	0-10
Having difficulty feeling relaxed or comfortable	2.6 (2.6)	0-10
Depressed	1.3 (2.2)	0-10
Anxious	1.6 (2.4)	0-10
Difficulties concentrating	1.7 (2.5)	0-9
Having difficulty taking care of my personal hygiene	2.9 (2.9)	0-10
Having difficulty returning to work or usual home activities	6.6 (3.4)	0-8
Pain in the surgical wound	4.6 (3.0)	0-10
Reddened surgical wound	1.5 (2.4)	0-10
Swollen surgical wound	2.1 (2.8)	0-10
Fever	0.3 (0.9)	0-4
Nausea, vomiting, or both	0.9 (2.0)	0-8
Dizziness	1.2 (2.0)	0-8
Headache	1.1 (1.9)	0-8

### Floor or Ceiling Effects

The distributions of the SwQoR-LA scores on days 1-7 were skewed to the left and ranged between 0 and 101. No patient gave the maximum score (ie, there was no ceiling effect). No floor effects were present either (Table 3).

**Table 3:** Response rate, mean, minimum and maximum scores, floor effect, Cronbach α, and split-half coefficient of the Swedish Web Version of the Quality of Recovery Scale Adapted for Patients Undergoing Local Anesthesia and Peripheral Nerve Blockade (SwQoR-LA) on postoperative days 1-7.

	Day 1 (n=95)	Day 2 (n=90)	Day 3 (n=93)	Day 4 (n=89)	Day 5 (n=85)	Day 6 (n=80)	Day 7 (n=78)
Response rate, %	88.8	84.1	86.9	83.2	79.4	74.8	72.9
SwQoR-LA, mean (SD)	35.7 (24.4)	27.0 (21.7)	20.5 (18.6)	20.0 (18.1)	18.0 (18.2)	16.8 (17.1)	15.5 (15.5)
SwQoR-LA, minimum-maximum	0-99	0-101	0-83	0-85	0-92	0-91	0-84
SwQoR-LA floor effect, n (%)	2 (2.1)	4 (4.4)	6 (6.4)	6 (6.7)	4 (4.7)	6 (7.5)	7 (8.9)
Cronbach α	.88	.87	.87	.87	.89	.88	.88
Split-half coefficient	0.86	0.80	0.81	0.85	0.81	0.82	0.86

### Validity

Construct validity analysis indicated low correlations between the SwQoR-LA on day 1 and PACU stay (rho=0.21, *P*=.05), duration of surgery (rho=0.28, *P*<.001), and patient age (rho=0.18, *P*=.11). There were no significant differences in global SwQoR-LA between the genders on day 1: women, 38.7 (SD 24.9) versus men, 29.8 (22.6).

Discriminant validity was determined by comparing 8 symptoms related to general anesthesia on postoperative day 1 between patients who had undergone general anesthesia and patients who had undergone local anesthesia. All symptoms except for “Diarrhea” were significantly lower in the patients who had undergone local anesthesia (Table 4).

**Table 4:** Discriminant validity of the Swedish Web Version of the Quality of Recovery Scale Adapted for Patients Undergoing Local Anesthesia and Peripheral Nerve Blockade (SwQoR-LA), as analyzed with Mann-Whitney U tests.

Item	General anesthesia (n=313), mean (SD)	Local anesthesia (n=95), mean (SD)	*P* value
Voice not sounding the same as usual	1.7 (2.7)	0.6 (1.7)	<.001
Sore throat	2.0 (2.8)	1.4 (1.2)	<.001
Sore mouth	1.0 (2.0)	0.1 (0.7)	<.001
Having trouble breathing	0.8 (1.7)	0.3 (1.2)	.02
Muscle pain	2.2 (2.8)	1.4 (2.2)	.01
Trouble urinating	1.0 (2.0)	0.4 (1.4)	.01
Feeling constipated	1.2 (2.3)	0.6 (1.6)	.01
Diarrhea	0.4 (1.2)	0.3 (1.0)	.32

### Reliability

Regarding internal consistency, the Cronbach α for the sum score of the SwQoR-LA ranged between .87 and .89, while the split-half coefficient ranged between 0.82 and 0.90 (Table 3).

### Responsiveness

Cohen *d* effect size between days 1 and 7 was 0.98. The mean change in the global SwQoR-LA score from day 1 to day 7 was –19.7 (SD 19.4) with a 95% CI of 15.2-24.2, *P*<.001. The percentage of change from baseline was 43.4%.

## Discussion

The aim of this study was to perform a psychometric evaluation of the use of a real-time recovery questionnaire for a population of day surgery patients undergoing local and peripheral block anesthesia, namely, the SwQoR-LA. To our knowledge, the SwQoR-LA is the first real-time recovery questionnaire that has been developed and tested for this specific group of patients. The SwQoR-LA was shown to have high validity, reliability, responsiveness, and clinical user friendliness. The construct validity of the SwQoR-LA was supported for PACU stay and duration of surgery, although there were low correlations. However, no significant correlations were found between age and SwQoR-LA. Strong correlations have been reported previously for patients undergoing major surgery [10,24-26]. However, due to the minor nature of the surgery and anesthesia in the present study, low correlations were expected. We found no differences between genders, which is in line with a study from Iceland [27] and an earlier publication of ours [18,28]. However, gender differences in postoperative recovery have been reported in earlier studies with inpatients undergoing surgery from Denmark [29], Iran [26], and Australia [24,25].

Discriminant validity was confirmed in 7 of the 8 symptoms that are mainly related to general anesthesia. The symptom that was not significant was “Diarrhea*,*” possibly due to the minor surgery procedures. However, a symptom that that seems to be missing in SwQoR-LA is postoperative fatigue. Postoperative fatigue has been reported as a common symptom after day surgery [30] and occurs in patients, irrespective of whether general or local anesthesia is used [1,30,31]. Postoperative fatigue has a large impact on patients’ daily life [31,32]. Postoperative symptoms such as early postoperative cognitive decline, [33] pain, anxiety, depression, stress, and changes in sleep patterns [34] seem to influence the severity of fatigue. However, if the symptom “Diarrhea” should be removed from SwQoR-LA in favor of the symptom “Fatigue” has to be further investigated as well as psychometrically evaluated.

Postoperative pain in the surgical wound is an important symptom to measure repeatedly and thereby identify its progression. In this study, pain in the surgical wound was the symptom that occurred most frequently, with an average level of 4.6 at day 1. The levels decreased to 3.3 on day 2 and to <3 on day 3 and thereafter. In a recent study by Rodrigues et al [35], 3.8% of the patients undergoing peripheral block and 2.1% of the patients undergoing local anesthesia suffered from uncontrolled pain on days 1-2. However, they did not assess the levels of postoperative pain as well as the progression over time.

Internal consistency of the SwQoR-LA showed acceptable values, with a Cronbach α range of .87-.88 for days 1-7. This result is in line with the SwQoR, for which Cronbach α ranges from .91 to .93 for days 1-7 [18]. Cronbach α is directly affected by scale length and increases with an increasing number of items [22]. Nevertheless, the length of the scale is not the only accurate judgment [36]. Nunnally and Bernstein [37] are frequently quoted for the following cut-off values: Cronbach α of at least .70 in the early stages of research; Cronbach α of .80 in an applied setting when cut-off scores are used and for basic research; and Cronbach α of .90 for scales used for clinical purposes, with a desired standard of .95 in such cases [36,37]. The SwQoR-LA should be concentrated on individual items and global scores—a recommendation that has also been made for the SwQoR [18]. Therefore, and because the sample size was too small (ie, <10 participants per item) [22], no factor analysis of the SwQoR-LA was performed.

The response rate on day 1 was 88.8% and decreased over time, with a response rate of 72.9% observed on day 7. This decreased response rate may reflect the fact that the symptoms were low on day 7, as the changes from baseline were 43.4%, from 37.7 on day 1 to 15.5 on day 7. This finding indicates that the SwQoR-LA has the ability to detect clinically important changes [22] following day surgery in patients undergoing local and peripheral block anesthesia. In an earlier study by the same research group, the patients considered that a period of 9 days was acceptable for assessing postoperative recovery after day surgery [17]. However, that population included both patients undergoing local anesthesia and those undergoing general anesthesia [17]. As well, both patients and clinicians using RAPP in clinical practice have pointed out that 14 days of assessment is too long for the short recovery period after minor surgery with local or peripheral block anesthesia. We therefore suggest that 7 days of postoperative assessment with the SwQoR-LA is appropriate for this group of patients. Furthermore, the SwQoR-LA is a real-time recovery, electronic assessment, which is important to identify specific deficits in patient recovery [12,13]. As postoperative recovery is a dynamic and individual process that includes physical, psychological, social, and habitual aspects [1-3], recovery assessments should be multidimensional, be patient focused, and occur in real time at multiple clinically relevant postoperative time points [14]. The ability to identify symptom-speciﬁc recovery failure and implement targeted therapies to improve recovery is an important goal for perioperative care [12,14]. This requires a real-time recovery questionnaire such as the SwQoR-LA for early identiﬁcation of recovery failure as well as for assessment of the outcomes following interventions in clinical practice and clinical trials [12]. If access to the web version of the SwQoR-LA is not possible, the paper version can be used instead, as there is equivalence between the web version and paper version [17].

### Limitations

There are some limitations in our study. First, the sample size is relatively small, but considered sufﬁcient for examining psychometric properties with 16 items. However, there is no consensus about the number of participants for each type of psychometric analysis. To analyze construct validity, responsiveness, and floor and ceiling effects, a sample size of at least 50 participants is recommended [22]. Second, no test-retest reliability was conducted. This feature could be improved in future studies by involving a larger pool of patients undergoing a wider range of peripheral nerve block. Third, the duration for data entry was not measured.

### Conclusions

To our knowledge, this study is the first to evaluate a real-time recovery questionnaire, the SwQoR-LA, in patients undergoing local or peripheral nerve block anesthesia. The SwQoR-LA is valid, reliable, responsive, and clinically feasible for the real-time assessment of patient recovery in order to detect those patients who might beneﬁt from timely follow-up and intervention.

## Abbreviations

COSMIN: COnsensus-based Standards for the selection of health Measurement INstruments
PACU: postanesthesia care unit
RAPP: Recovery Assessment by Phone Points
SwQoR-LA: Swedish Web Version of the Quality of Recovery Scale Adapted for Patients Undergoing Local Anesthesia and Peripheral Nerve Blockade
